# Serotonergic Innervations of the Orbitofrontal and Medial-prefrontal Cortices are Differentially Involved in Visual Discrimination and Reversal Learning in Rats

**DOI:** 10.1093/cercor/bhaa277

**Published:** 2020-10-12

**Authors:** Johan Alsiö, Olivia Lehmann, Colin McKenzie, David E Theobald, Lydia Searle, Jing Xia, Jeffrey W Dalley, Trevor W Robbins

**Affiliations:** Department of Psychology, Behavioural and Clinical Neuroscience Institute, University of Cambridge, Cambridge CB2 3EB, UK; Department of Psychology, Behavioural and Clinical Neuroscience Institute, University of Cambridge, Cambridge CB2 3EB, UK; Department of Psychology, Behavioural and Clinical Neuroscience Institute, University of Cambridge, Cambridge CB2 3EB, UK; Department of Psychology, Behavioural and Clinical Neuroscience Institute, University of Cambridge, Cambridge CB2 3EB, UK; Department of Psychology, Behavioural and Clinical Neuroscience Institute, University of Cambridge, Cambridge CB2 3EB, UK; Department of Psychology, Behavioural and Clinical Neuroscience Institute, University of Cambridge, Cambridge CB2 3EB, UK; Department of Psychology, Behavioural and Clinical Neuroscience Institute, University of Cambridge, Cambridge CB2 3EB, UK; Department of Psychiatry, University of Cambridge, Cambridge CB2 0SZ, UK; Department of Psychology, Behavioural and Clinical Neuroscience Institute, University of Cambridge, Cambridge CB2 3EB, UK

**Keywords:** cognitive flexibility, medial prefrontal cortex, orbitofrontal cortex, serotonin, translational neuroscience

## Abstract

Cross-species studies have identified an evolutionarily conserved role for serotonin in flexible behavior including reversal learning. The aim of the current study was to investigate the contribution of serotonin within the orbitofrontal cortex (OFC) and medial prefrontal cortex (mPFC) to visual discrimination and reversal learning. Male Lister Hooded rats were trained to discriminate between a rewarded (A+) and a nonrewarded (B−) visual stimulus to receive sucrose rewards in touchscreen operant chambers. Serotonin was depleted using surgical infusions of 5,7-dihydroxytryptamine (5,7-DHT), either globally by intracebroventricular (i.c.v.) infusions or locally by microinfusions into the OFC or mPFC. Rats that received i.c.v. infusions of 5,7-DHT before initial training were significantly impaired during both visual discrimination and subsequent reversal learning during which the stimulus–reward contingencies were changed (A− vs. B+). Local serotonin depletion from the OFC impaired reversal learning without affecting initial discrimination. After mPFC depletion, rats were unimpaired during reversal learning but slower to respond at the stimuli during all the stages; the mPFC group was also slower to learn during discrimination than the OFC group. These findings extend our understanding of serotonin in cognitive flexibility by revealing differential effects within two subregions of the prefrontal cortex in visual discrimination and reversal learning.

## Introduction

The indoleamine neurotransmitter serotonin (5-hydroxytryptamine; 5-HT) contributes to various aspects of cognition in both humans and animals, including memory processing (Mendelsohn et al. 2009; Bekinschtein et al. 2013), impulse control (Worbe et al. 2014; Fonseca et al. 2015), reinforcement learning (den Ouden et al. 2013; Iigaya et al. 2018), and behavioral flexibility (Evers et al. 2007; Barlow et al. 2015). Acute tryptophan depletion (ATD) transiently lowers brain 5-HT by reducing its synthesis (Stancampiano et al. 1997; Young et al. 1999; Crockett et al. 2012) and impairs reversal learning (Rogers et al. 1999), a form of cognitive flexibility that implicates the orbitofrontal cortex (OFC; Jones and Mishkin 1972; Dias et al. 1996; Chudasama and Robbins 2003; Izquierdo et al. 2017). Further understanding of the functions of prefrontal 5-HT in reversal learning will assist in the development of cognitive enhancers for psychiatric disorders linked to reversal-learning deficits, including obsessive–compulsive disorder (Chamberlain, Fineberg, et al. 2006a), schizophrenia (Leeson et al. 2009), and cocaine use disorder (Ersche et al. 2008).

Animal experiments have confirmed the importance of 5-HT for behavioral flexibility and refined our understanding of serotonergic transmission in reversal learning. Boosting 5-HT tone by systemic treatment with either serotonin reuptake inhibitors (e.g., citalopram) or agents blocking monoamine oxidase A (e.g., moclobemide) improves reversal learning in rats and mice (Bari et al. 2010; Brigman et al. 2010; Barlow et al. 2015; Zhukovsky et al. 2017). Similar improvements have been reported in mouse models where elevated 5-HT tone has been induced genetically, e.g., by knock-out of the serotonin transporter gene *Slc6a4* (Brigman et al. 2010). Clarke and colleagues used local infusions of 5,7-dihydroxytryptamine (5,7-DHT) to show that selective prefronto-cortical (Clarke et al. 2004) or OFC (Clarke et al. 2007) 5-HT depletions in marmoset monkeys impair visual reversal learning but not another form of flexible behavior, extradimensional set-shifting (Clarke et al. 2005).

Studies investigating the effects of reduced serotonergic transmission on cognitive flexibility in rodents have yielded more equivocal results. Para-chlorophenylalanine (PCPA)—a selective and irreversible inhibitor of tryptophan hydroxylase—impaired reversal learning in a bowl-digging task for rats, whereas both discrimination learning and set-shifting was intact (Lapiz-Bluhm et al. 2009; Wallace et al. 2014). However, Brigman et al. found no impairments in a touchscreen visual discrimination and reversal task for mice after reduced 5-HT levels using either knock-out of the *Pet1* gene or systemic PCPA treatment (Brigman et al. 2010). Similarly, Izquierdo and colleagues reported that a medium dose of PCPA in rats had no effect on either visual discrimination or subsequent reversal learning, whereas a higher dose caused a severe impairment in the pretraining acquisition stages of the touchscreen protocol (Izquierdo et al. 2012). Masaki et al. (2006) reported impairments in both acquisition and reversal in a go/no-go task for rats after parachloroamphetamine treatment, in agreement with reports of impaired acquisition and performance in go/no-go task after global 5,7-DHT lesions (Harrison et al. 1999).

One potential confound in the rodent literature has been the use of global serotonergic manipulations when 5-HT has disparate functions in cognitive control within and across cortical and subcortical areas (Fletcher et al. 2009). For instance, intra-OFC, but not intra-mPFC, 5-HT_2C_ receptor antagonism improves spatial reversal learning in rats (Boulougouris and Robbins 2010) and local lateral OFC 5-HT_2C_ receptor antagonism also improves performance on a touchscreen visual reversal-learning task (Alsiö et al. 2015). We and others have previously used 5,7-DHT to deplete 5-HT either globally via intracerebroventricular (i.c.v.) infusions or after local infusions into defined brain areas in rats and marmoset monkeys. This approach allows profound and localized tissue 5-HT depletion. However, 5-HT systems can recover after localized 5,7-DHT lesions (Man et al. 2010; Rygula et al. 2015) and normal baseline levels of extracellular 5-HT may remain even though only a small proportion of fibers are spared by such treatment (Kirby et al. 1995).

Here, we employed 5,7-DHT infusions to investigate the effect of 5-HT depletion on visual discrimination and reversal learning in the rat. We first compared the effects of global 5-HT depletion in rats that received i.c.v. infusions of 5,7-DHT either before the acquisition of visual discrimination (Experiment 1) or after such training, but prior to the reversal stages of the test (Experiment 2). We then investigated the effects of local 5,7-DHT infusions into either the OFC or mPFC on both discrimination and reversal learning by lesioning the rats prior to visual discrimination testing (Experiment 3). A separate group of rats was infused with 5,7-DHT into the OFC or mPFC and used for neurochemical analyses three weeks after surgery, to investigate the extent of 5-HT depletion at a time point equivalent to the start of behavioral testing on the reversal-learning stage.

## Materials and Methods

### Subjects

All subjects were male Lister Hooded rats (Charles River, UK), housed in pairs in a temperature-controlled room (22 °C), under diurnal conditions (12 h light/12 h dark; light on at 7 AM). The animals were 3 months of age at the start of behavioral training. Rats were food deprived and maintained at 85% of their free-feeding weight throughout the experiment, except during postsurgical recovery (see below). All testing occurred at a regular time during the light period. All experimental procedures were carried out under UK Home Office Project licenses PPL 80/1324, 80/1767 and 70/7548 in accordance with the UK Animals (Scientific Procedures) Act 1986 and local ethical approval by the University of Cambridge Animal Welfare and Ethical Review Body (AWERB).

### Surgery

**Figure 1:**
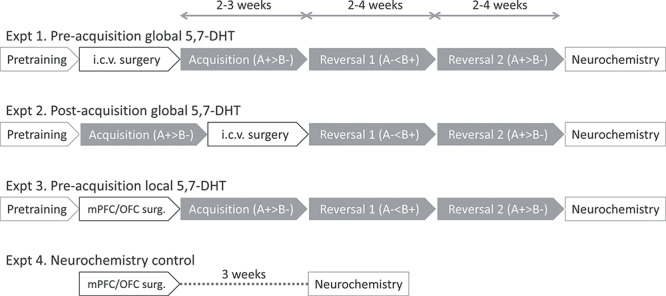
Experimental time line for Experiments 1–4. Experiments 1–3 were behavioral cohorts and the cohort in Experiment 4 was used for neurochemical validation of the 5-HT depletion locally in the mPFC and OFC only. Note that the order of visual discrimination training (acquisition) and surgery is different in Experiment 1 and Experiment 2.

Figure 1 depicts the experimental timelines. All rats were treated 30 min before the start of surgery with desipramine HCl (20 mg/mL/kg, i.p.) dissolved in double-distilled water (Sigma Chemical Co.) to protect noradrenergic neurons from the neurotoxin. For experiments 1–3, rats were anaesthetized by an intraperitoneal injection of Avertin (10 g 2,2,2-tribromoethanol (Sigma) in 5 g tertiary amyl alcohol, diluted in a solution of 40 mL ethanol and 450 mL phosphate-buffered saline) given at a dose of 1 mL/100 g, and placed in a stereotaxic frame fitted with atraumatic bars (David Kopf Instruments). For experiment 4, rats were anaesthetized with isoflurane (induction at 5% and maintenance at 2–3%). For all surgeries, the incisor bar was set at −3.3 mm relative to the interaural line in the flat skull position. The scalp was retracted to expose the skull and craniotomies were made directly above the target region of the brain (according to Paxinos and Watson, 1997). I.c.v. and local mPFC and OFC infusions were made using a 31-gauge, nonbeveled stainless steel injector (Cooper Needleworks).

For i.c.v. lesions, bilateral infusions of 80 μg/10 μL 5,7-DHT creatine sulphate (80 μg free base dissolved in 10 μL of 0.1% ascorbic acid in saline) were made over 8 min after which the injector was left in situ for 2 min before withdrawal to allow diffusion of the toxin. The following coordinates were used: AP: −0.9 mm from bregma, ML: ±1.5 mm from the midline and DV: −3.5 mm from dura. Sham rats received vehicle infusions (10 uL/side) instead of toxin.

For OFC and mPFC lesions, two bilateral injections of 2 μg/0.5 μL 5,7-DHT creatine sulfate dissolved in 0.1% ascorbic acid in saline were made at a rate of 3 min per injection after which the injector was left in situ for 3 min before withdrawal. For OFC lesions, the following coordinates were used: AP: +4.20 mm from bregma, ML: ±3.0 mm from midline; DV: −3.0 mm from dura (0.5 μL) and ML: ±1.0 mm, DV: −3.4 mm (0.5 μL). For PFC lesions, the following coordinates were used: AP: +3.20 mm from bregma, ML: ±0.6 mm from midline, DV: −3.4 mm from dura (0.5 μL) and AP: +2.70 mm, ML: ±0.6 mm, DV: −3.6 mm (0.5 μL). Animals allocated to the sham-lesioned group received the same surgical treatment as OFC or PFC groups but received infusions of vehicle (0.1% ascorbic acid in 0.9% saline) instead of toxin (0.5 μL per infusion, as above).

Following surgery, animals in Experiments 1–3 were given free access to food for ≥5 days before behavioral testing to allow for the degeneration of serotonergic neurons (cf., Eagle et al. 2009). Animals in the neurochemistry cohort (Experiment 4) were kept on free food throughout the experiment and received meloxicam subcutaneously (1 mg/kg) at the start of the surgery, as well as orally (0.6 mg/kg) during three days after the surgery.

### Apparatus

**Figure 2:**
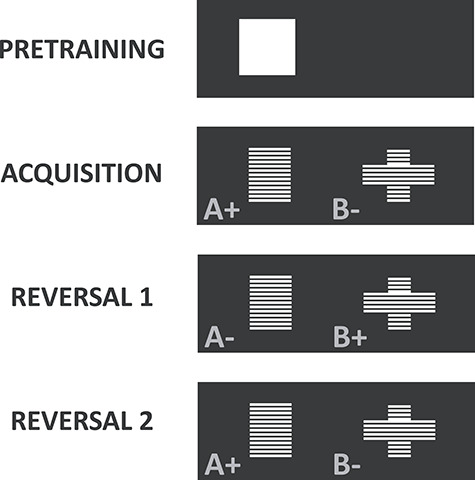
Stimulus contingencies during the acquisition of visual discrimination, reversal 1 and reversal 2. Stimuli assigned to reward (+) and nonreward (−) were counterbalanced (rectangle and cross) across groups.

The apparatus has been described previously (Bussey et al. 1994; Bussey et al. 1997; Chudasama et al. 2001; Chudasama and Robbins 2003). Testing took place in six automated touch-screen chambers (Cambridge Cognition) housed within a sound-attenuating box fitted with a fan for ventilation and the masking of extraneous noise. The inner chamber (45 × 30 × 30 cm) consisted of three aluminum walls and one Perspex wall incorporating the door, an aluminum perforated floor and a clear Perspex ceiling. A 3-W house light was attached to a side wall. Located centrally at the rear of the chamber was a food magazine attached to a pellet dispenser (45 mg sucrose pellets, Noyes dustless pellets, Sandown Scientific) and equipped with a 3-W panel light and photocells to allow detection of magazine entries. At the front of the chamber was a monitor (Intasolve) surrounded by an array of infrared beams that ran across the surface of the display. Contact with the screen was detected by the beams being broken, making the screen touch-sensitive. A black Perspex “mask” was attached to the face of the display unit 2 cm away from its surface to block access to the display unit except through two response windows (8 × 9 cm) located at 15 cm from the floor of the chamber. A shelf extended 6.5 cm from the surface of the mask supported by springs was positioned just beneath the response windows to help the rats to rear up to touch the screen. In these response windows, separated by a black divider to prevent from accidental approaches to the adjacent response window, the stimuli were presented: white grid patterns in the shape of a rectangle and a cross that were equated for luminance (Chudasama et al. 2001; Chudasama and Robbins 2003). See Figure 2.

#### Pretraining

Subjects were initially given one 30-min session of habituation to the testing chamber with the magazine filled with pellets and the magazine and house lights on. They were then given 30-min sessions in which they were shaped to collect food pellets delivered to the magazine on a random interval 40-s schedule with the magazine light signaling a pellet delivery. Once the animals were reliably retrieving a minimum of 50 pellets per session, they were trained to respond to stimuli presented on the touch screen. In this procedure, a large white square (filling the response window) was randomly presented in one of the two response windows. The square remained on the screen until the rat responded to it, after which the rat was rewarded with a pellet, signaled by the magazine light. Once the rat was able to obtain 50 reinforcements within 20 min, the acquisition of the visual discrimination commenced.

In Experiments 1 and 3, surgery was performed after the touch-training sessions and groups were matched according to the number of sessions to reach the criterion. After the postsurgical recovery period, rats were briefly re-trained on the touch-training procedure to ensure adequate performance prior to the start of acquisition (see Fig. 1).

#### Acquisition

For each rat, one stimulus was designated A+; the other was designated B− (see Fig. 2). The stimulus contingencies were counterbalanced across groups. The same pair of stimuli was presented on every trial and the configuration for each trial (which stimulus was on the left and which one was on the right) was determined pseudo-randomly (except during correction trials, see below). The session began with the delivery of a pellet as well as the illumination of the houselight and the magazine light. A nosepoke in the magazine extinguished the magazine light and initiated a 5-s ITI after which the stimuli were presented. The rat was then required to make a response by selecting a stimulus by touching the screen with a nosepoke. The stimuli were presented until the rat responded to either one of them. A correct response to the A+ was followed by the disappearance of the stimuli and the delivery of a pellet concomitant with the illumination of the food magazine. An incorrect response to the B− stimulus resulted in the disappearance of the stimuli and the house light being extinguished for a timeout period of 5 s. When the rat had collected the reward after a correct response, or after the timeout period had passed after an incorrect response, it was required to wait for the ITI before the next trial commenced automatically with the presentation of the stimuli. Each session consisted of 101 trials, but as part of a correction procedure, after an incorrect choice, rats received additional “correction trials” in which the same stimulus configuration (A+ and B− remained in the same left/right locations) was presented over successive trials until the rat had responded correctly. All animals were required to learn the correct, reinforced stimulus to a criterion of 85% correct response (in noncorrection trials) in a session after which they moved to the next stage.

#### Retention

In Experiment 2, the i.c.v. lesion was made after rats had acquired the visual discrimination. Consequently, an additional stage for these rats consisted of testing the retention of the initial discrimination learnt during the acquisition stage. Once the subjects exhibited 85% correct responding, they were moved to the reversal stages. Rats required 1–3 retention sessions to reach this criterion.

#### Reversals

The reward contingency was reversed such that the previously nonrewarded stimulus was now the correct reinforced stimulus (i.e., A+ became A− and B− became B+). Animals were required to reach the criterion of 85% correct responses. For the second reversal, the reward contingencies were reversed again, leading back to the contingencies of the acquisition stage (A+/B−). The behavioral experiment ended once animals reached 85% correct responses.

### Performance Measures

On the basis on noncorrection trials only, other measures were calculated: average correct and incorrect response latency, which was the time from the stimulus onset to the time the rat made a response on rewarded and nonrewarded trials, respectively, and average magazine latency, which was the time from a correct response to the time the rat entered the magazine to collect the reward. Percentage side bias was also measured, calculated arbitrarily as the number of responses to the left response window divided by the total number of trials for that session. The side bias measure did not differ significantly from chance (50%) for any of the experiments.

### Data Analysis

Some rats were excluded from the study for the following reasons (see Table 1). In Experiment 1, one sham-operated rat died during recovery and two rats were removed from testing due to the development of audiogenic epileptic seizures, which is a known occurrence in the Lister Hooded strain (Commissaris et al. 2000). In the 5,7-DHT i.c.v. group, one rat failed to finish the behavioral testing and another rat presented a unilateral 5-HT depletion and these animals were excluded from the study. Thus, the final group numbers for this experiment was sham *n* = 8 and 5,7-DHT *n* = 9. In Experiment 2, two rats were removed from the sham group during testing due to the development of epileptic seizures and, in the 5,7-DHT i.c.v. group, one rat died during surgery and a second died due to postsurgical complications. Thus, the final group number for this experiment was as follows: Sham, *n* = 8; i.c.v., *n* = 8. In Experiment 3, rats that had received OFC and mPFC vehicle infusions were combined into one sham group (by design); initial inspection showed no differences in any of the main measures i.e., sessions or errors to criterion in any of the stages (visual discrimination, reversal 1 and reversal 2) between sham sites. One sham animal and two 5,7-DHT OFC rats were excluded due to the development of epileptic seizures. Two rats in the 5,7-DHT OFC group failed to finish the behavioral testing. In the 5,7-DHT mPFC group, one rat died during surgery, a second one later due to postsurgical complications, and two other rats were removed from testing due to the development of epileptic seizures. Thus, the final group number for this experiment was as follows: sham, *n* = 9; OFC, *n* = 6; mPFC, *n* = 5. No rats were excluded from the neurochemistry control study (Expt. 4): mPFC shams, *n* = 4; OFC shams, *n* = 4; OFC 5,7-DHT, *n* = 6, mPFC 5,7-DHT, *n* = 6. It should be noted that all rats were routinely monitored multiple times per day and that none of the remaining rats displayed any signs of epileptic seizures.

**Table 1:** Experimental subjects per experiment and group

Cohort	Group	Initial *n*	Final *n*
Expt 1. I.c.v. 5,7-DHT preacquisition	i.c.v. 5,7-DHT	11	9
	Sham	11	8
Expt 2. I.c.v. 5,7-DHT postacquisition	i.c.v. 5,7-DHT	10	8
	Sham	10	8
Expt 3. Local 5,7-DHT – behavioral cohort	mPFC 5,7-DHT	9	5
	OFC 5,7-DHT	10	6
	Sham	10	9
Expt 4. Local 5,7-DHT – neurochemistry cohort	mPFC 5,7-DHT	5	5
	mPFC sham	4	4
	OFC 5,7-DHT	6	6
	OFC sham	4	4

Note: See main text for exclusion criteria.

Data from the remaining animals were analyzed using repeated-measures analysis of variance (ANOVA) within the SPSS statistical package, version 26 (SPSS Inc.). Homogeneity of variance across testing stages was assessed by the Mauchly Sphericity Test and Greenhouse–Geisser correction was used when appropriate. The distribution of residuals was inspected using histograms and Q-Q plots, and analyses violating the assumptions of normality of distribution were square root or log transformed. Where appropriate, post hoc comparisons were conducted using one-way ANOVA or the Fisher’s LSD test. In Experiment 1 (preacquisition), the between-subjects factor was lesion (two levels: Sham and i.c.v.) and the within-subject factor was stage (three levels: Acquisition, Reversal 1 and Reversal 2). In Experiment 2 (post acquisition), the between-subjects factor was lesion; retention was analyzed separately by Student’s *t*-tests and performance during reversals was analyzed with stage as the within-subject factor (two levels: Reversal 1 and Reversal 2). This approach was chosen as the variability during retention was very low. For Experiment 3, the between-subjects factor was lesion (three levels: Sham, OFC and mPFC) and the within-subject factor was stage (three levels: Acquisition, Reversal 1 and Reversal 2).

### Postmortem Neurochemical Assessment

At the end of each experiment, animals were killed through exposure to increasing concentrations of carbon dioxide. Brains were rapidly extracted and frozen on dry ice. Coronal sections were cut (150 μm thickness) on a cryostat (−10 °C) from the frontal pole to the hippocampus and mounted onto prechilled microscope slides. Slides were at −80 °C before further dissection. A stainless-steel micro-punch (0.75 mm diameter) and a razor blade were used to remove 0.4–2-mg aliquots of tissue from the following left and right brain regions on frozen slides: medial prefrontal and orbitofrontal cortices, nucleus accumbens, amygdala, and the dorsal hippocampus. Aliquots were weighed, and samples were homogenized in 100 μL of 0.2 M perchloric acid to precipitate protein material. Following centrifugation at 6000 rpm for 20 min at 4 °C, 25 μL supernatant was taken and placed into autoinjector microvials ready for analysis. Levels of serotonin (5-HT), noradrenaline (NA), and dopamine (DA) were determined in brain samples by reverse-phase high-performance liquid chromatography (HPLC) as described elsewhere (Dalley et al. 2002; Rygula et al. 2015). Due to technical issues it was not possible to measure DA in Experiments 1, 2, and 3.

Note that whereas neurochemistry data are presented from the behavioral cohorts from the global 5-HT depleted rats (i.c.v. 5,7-DHT) and their controls in Experiments 1 and 2, an additional cohort, killed three weeks after surgery, was used to estimate the degree of 5-HT depletion after local infusion of 5,7-DHT into the mPFC and OFC in Experiment 3. This approach was used as it has been documented that 5-HT innervation in rats (Kirby et al. 1995) and marmosets (Man et al. 2010) recovers over time after 5,7-DHT treatment, making contemporaneous assessment of depletion during the behaviorally critical time points difficult. Indeed, the behavioral cohort, killed after the training was ceased for all rats in Experiment 3, showed full or partial recovery of 5-HT in the OFC and mPFC, respectively. Whereas neurochemistry results are presented as percentages of control (sham) values, all statistics (Student’s *t*-tests) were performed on the raw data. This prevents inflated differences where 5-HT levels were not detected (and therefore set to zero) in 5,7-DHT rats. One-tailed *t*-tests were employed to evaluate the predicted depletion of 5-HT levels in target areas, whereas two-tailed t-tests were used for off-target 5-HT levels and all noradrenaline measurements.

## Results

### Experiment 1: Effects of Preacquisition Intracerebroventricular 5,7-DHT on Visual Discrimination Learning and Reversal

#### Neurochemical Findings

**Figure 3:**
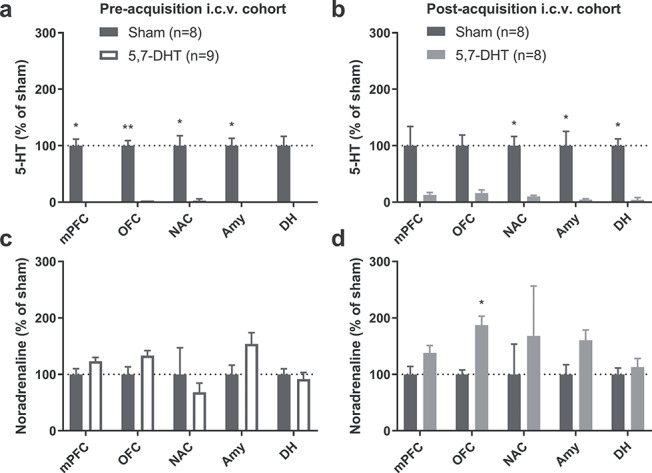
Intracerebroventricular (i.c.v.) infusions of 5,7-DHT caused profound reductions in 5-HT throughout the brain in Expt. 1 (preacquisition cohort) and Expt. 2 (postacquisition cohort). (*a*, *b*) The loss of 5-HT was on average more than 80% in cortical and subcortical areas in both the preacquisition (*a*) and postacquisition (*b*) cohorts. (*c*, *d*) There were no indications of noradrenaline depletion in either the preacquisition (*c*) or the postacquisition (*d*) cohort; instead, there was evidence of an upregulated noradrenaline system in the postacquisition groups. mPFC, medial prefrontal cortex; OFC, orbitofrontal cortex; NAC, nucleus accumbens; Amy, amygdala; DH, dorsal hippocampus. ^*^P < 0.05; ^*^^*^P < 0.01; Student’s *t*-test, one-tailed to detect 5-HT depletion and two-tailed to test for effects on noradrenaline.

Rats received 5,7-DHT infusions after initial touchscreen training but prior to the acquisition of the visual discrimination. Postmortem analyses of monoamine concentrations throughout the forebrain revealed a profound 5-HT depletion of more than 95% in i.c.v. 5,7-DHT-treated animals as compared to sham-operated controls (Fig. 3). The depletion reached statistical significance in the mPFC, OFC, NAc, and amygdala (Fig. 3*a*). Levels of noradrenaline were increased in some regions by i.c.v. 5,7-DHT infusions, albeit not significantly so (Fig. 3*c*).

#### Effect of Global 5-HT Depletion on Visual Discrimination and Reversal Learning

**Figure 4:**
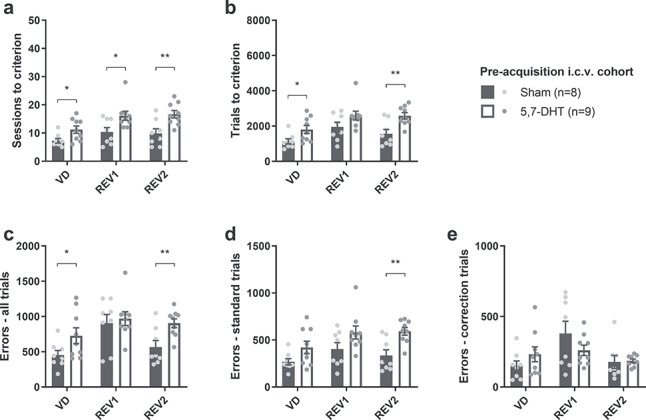
Global 5-HT depletion impaired both visual discrimination and subsequent reversal learning when 5,7-DHT was infused intracerebroventricularly (i.c.v.) prior to the initial acquisition of stimulus–reward contingencies (Expt. 1). (*a*) Sessions required to reach criterion (85% correct) in each stage for the preacquisition experiment. (*b*) Total trials completed until reaching training criterion for each phase. (*c*) Total number of errors committed up to and including the session in which criterion was reached. (*d*, *e*) Errors committed on (*d*) standard trials and (*e*) correction trials until reaching criterion. Fisher’s LSD analysis after significant main effect of treatment: ^*^P < 0.05; ^*^^*^P < 0.01.

Forebrain 5-HT depletion impaired both acquisition and reversal of visual discrimination (Fig. 4). Overall, across learning stages, the forebrain 5-HT depleted rats required more sessions relative to shams to reach learning criterion (square root transformed; *F*_1,15_ = 16.4; *P* = 0.001; Fig. 4*a*), but there was no significant lesion × stage interaction (*F*_2,30_ = 0.52; *P* = 0.60). Pairwise comparisons revealed that rats in the 5,7-DHT group needed more sessions to reach criterion in each stage of the task: visual discrimination (*P* = 0.022), reversal 1 (*P* = 0.016) and reversal 2 (*P* = 0.005). Global 5-HT depletion also affected the number of trials required to reach the learning criterion (square root transformed; main effect of lesion: *F*_1,15_ = 12.3; *P* = 0.003; Fig. 4*b*), an effect that did not depend on stage (lesion × stage interaction: *F*_2,30_ = 0.66; *P* = 0.53). Pairwise comparisons showed a significant effect of lesion during acquisition (*P* = 0.025) and reversal 2 (*P* = 0.004), whereas no significance was detected for reversal 1 (*P* = 0.081).

On the number of errors to criterion, there was also a main effect of lesion (log transformed; *F*_1,15_ = 9.40; *P* = 0.008; Fig. 4*c*) and no significant lesion × stage interaction (*F*_2,30_ = 1.50; *P* = 0.26). Pairwise comparisons revealed a significant difference between 5-HT depleted rats and shams during the acquisition of the visual discrimination (*P* = 0.048) and during the second reversal (*P* = 0.005).

The types of errors were further broken down into errors committed on correction trials (“correction errors”) and errors on standard, noncorrection trials. The deficit was clearer when considering only noncorrection-trial errors: 5-HT depleted rats committed more errors overall (square root transformed; main effect of lesion: *F*_1,15_ = 12.7; *P* = 0.003; Fig. 4*d*). The lesion × stage interaction was again not significant (*F*_2,30_ = 0.63; *P* = 0.54). Pairwise comparisons revealed a significant difference only during reversal 2 (*P* = 0.003). On correction trials (Fig. 4*e*), in contrast, there was no significant main effect of lesion (*F*_1,15_ = 0.78; *P* = 0.39) but a significant lesion × stage interaction (*F*_2,30_ = 3.70; *P* = 0.037). Whereas the pairwise comparisons between the lesion groups failed to reach statistical significance, this interaction appeared to be driven by a decreased number of correction errors during the first reversal in the 5,7-DHT group and elevated numbers during the other two stages (Fig. 4*e*).

The impairment in reversal learning was accompanied in the 5,7-DHT-treated rats by faster correct response latencies (log transformed; main effect of lesion: *F*_1,15_ = 8.72; *P* = 0.010; Table 2), significant at each stage (acquisition: *P* = 0.045; reversal 1: *P* = 0.004; reversal 2: *P* = 0.030). The lesion × stage interaction failed to reach statistical significance (*F*_2,30_ = 2.92; *P* = 0.069). The latency to respond on incorrect trials was also reduced by the 5-HT depletion (main effect of lesion: *F*_1,15_ = 6.75; *P* = 0.020; lesion × stage interaction: *F*_2,30_ = 1.80; *P* = 0.18), significant at acquisition (*P* = 0.008) and reversal 1 (*P* = 0.005) but not reversal 2 (*P* = 0.36). In addition, lesioned animals were faster at collecting food rewards (main effect of lesion: *F*_1,15_ = 7.24; *P* = 0.017), an effect that did not depend on stage (lesion × stage interaction with Greenhouse–Geisser correction *F*_1.25,21.8_ = 0.048; *P* = 0.91). Pairwise comparisons revealed significant differences at each stage: acquisition (*P* = 0.023), reversal 1 (*P* = 0.026) and reversal 2 (*P* = 0.023). There was no difference in the side bias on standard trials (main effect of lesion: *F*_1,15_ = 0.001; *P* = 0.98; lesion × stage interaction: *F*_2,30_ = 1.45; *P* = 0.25; Table 2).

**Table 2:** Latencies and side bias in behavioral experiments

Cohort	Correct latency	Incorrect latency	Magazine latency	Side bias (left)
*Experiment 1 Pre-acquisition*				
Visual discrimination				
Sham 5,7-DHT	4334 ± 327 3493 ± 193^*^	4266 ± 256 3324 ± 175^*^	1816 ± 74 1571 ± 63^*^	48.36 ± 1.97 44.99 ± 1.30
Reversal 1				
Sham 5,7-DHT	4137 ± 534 2625 ± 126^*^	4027 ± 540 2630 ± 132^*^	1703 ± 83 1454 ± 60^*^	44.50 ± 4.38 44.52 ± 1.74
Reversal 2				
Sham 5,7-DHT	3805 ± 551 2669 ± 149^*^	3853 ± 598 3223 ± 329^*^	1710 ± 96 1446 ± 49^*^	43.96 ± 2.11 46.19 ± 1.68
*Experiment 2 Postacquisition*				
Retention				
Sham 5,7-DHT	3565 ± 317 3689 ± 365	6337 ± 2915 4592 ± 756	1590 ± 101 1828 ± 129	52.46 ± 1.58 47.72 ± 2.51
Reversal 1				
Sham 5,7-DHT	3795 ± 314 3864 ± 628	4074 ± 395 4196 ± 828	1605 ± 111 1707 ± 74	48.87 ± 2.39 51.19 ± 1.85
Reversal 2				
Sham 5,7-DHT	3422 ± 214 2936 ± 271	3736 ± 356 3876 ± 1074	1554 ± 101 1640 ± 81	45.50 ± 3.76 50.70 ± 2.28
*Experiment 3 Local depletions*				
Visual discrimination				
Sham OFC mPFC	4340 ± 439 5489 ± 461 5724 ± 690^*^^*^	4359 ± 452 5411 ± 525 4987 ± 372^*^^*^^*^	1595 ± 50 1626 ± 61 1546 ± 46	50.34 ± 2.47 49.19 ± 2.64 46.80 ± 2.58
Reversal 1				
Sham OFC mPFC	3543 ± 253 3507 ± 175 4761 ± 339^*^^*^	3590 ± 233 3555 ± 148 5201 ± 264^*^^*^^*^	1564 ± 47 1726 ± 90 1645 ± 85	46.62 ± 2.25 47.19 ± 3.82 44.14 ± 4.87
Reversal 2				
Sham OFC mPFC	3406 ± 134 3669 ± 538 4785 ± 363^*^^*^	3355 ± 148 3694 ± 526 4897 ± 343^*^^*^^*^	1586 ± 60 1647 ± 94 1562 ± 54	46.33 ± 2.41 45.98 ± 2.83 43.52 ± 5.77

Notes: All data are shown as mean ± SE; latencies are in milliseconds and side bias as percentage of bias arbitrarily to the left side on the screen. ^^*^^P < 0.05, main effect of lesion in a two-way ANOVA; ^^*^^*^^P < 0.05, ^^*^^*^^*^^P < 0.01, LSD post hoc mPFC versus Sham after significant main effect of lesion detected in a two-way ANOVA.

### Experiment 2: Effects of Postacquisition Intracerebroventricular 5,7-DHT on Visual Discrimination Retention Performance and Reversal

#### Neurochemistry

Rats in this group received 5,7-DHT or vehicle infusions after the acquisition of the visual discrimination, but prior to the first reversal of the stimulus–reward contingencies. Levels of 5-HT were strongly reduced (>80% depletion) across all brain regions investigated (Fig. 3*b*). The depletion was statistically significant in the NAc, amygdala, and dorsal hippocampus. Noradrenaline levels, in contrast, tended to be elevated across the regions analyzed (Fig. 3*d*); significantly elevated noradrenaline levels were observed in the OFC.

#### Behavioral Findings

**Figure 5:**
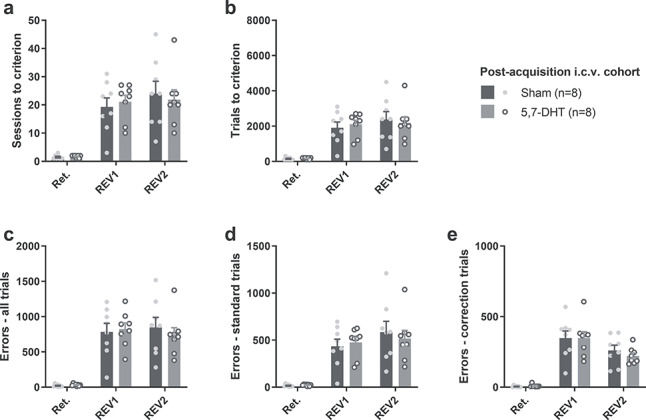
No effect of global 5-HT depletion when 5,7-DHT was infused after the initial visual discrimination training (Expt. 2). (*a*) Sessions required to reach criterion (85% correct) in each phase. (*b*) Count of trials up to and including the session in which criterion was reached. (*c*–*e*) Errors committed until criterion was reached: (*c*) total errors; (*d*) errors on standard (noncorrection) trials; (*e*) errors on correction trials.

When i.c.v. 5,7-DHT treatment was performed after the rats had reached criterion on the visual discrimination stage, there were no significant effects on subsequent choice performance either at the retention stage or the two reversals (Fig. 5). During retention, there was no effect of lesion on sessions (square root transformed; *t*_14_ = −0.95; *P* = 0.36; Fig. 5*a*) or trials (square root transformed; *t*_14_ = −0.95; *P* = 0.36; Fig. 5*b*) required to reach the 85% criterion, and no difference in the number of total errors (square root transformed; *t*_14_ = −1.59; *P* = 0.14; Fig. 5*c*), standard (noncorrection) errors (square root transformed; *t*_14_ = −1.36; *P* = 0.20; Fig. 5*d*), or correction errors (square root transformed; *t*_14_ = 1.66; *P* = 0.12; Fig. 5*e*) committed during these sessions. There were likewise no effects of lesion during the reversal stages on sessions (main effect: *F*_1,14_ = 0.019; *P* = 0.89; interaction *F*_1,14_ = 1.59; *P* = 0.23; Fig. 5*a*), trials (main effect: *F*_1,14_ = 0.019; *P* = 0.89; interaction *F*_1,14_ = 1.50; *P* = 0.24; Fig. 5*b*), total errors (main effect: *F*_1,14_ = 0.003; *P* = 0.96; interaction *F*_1,14_ = 1.29; *P* = 0.28; Fig. 5*c*), standard-trials errors (main effect: *F*_1,14_ = 0.004; *P* = 0.95; interaction: *F*_1,14_ = 1.80; *P* = 0.20; Fig. 5*d*) or correction errors (main effect: *F*_1,14_ = 0.097; *P* = 0.76; interaction: *F*_1,14_ = 0.46; *P* = 0.51; Fig. 5*e*). There was also no effect of lesion on the latency to respond on correct trials (log transformed; main effect: *F*_1,14_ = 0.274; *P* = 0.61; interaction: *F*_2,28_ = 1.32; *P* = 0.28; Table 2) or incorrect trials (log transformed; main effect: *F*_1,14_ = 0.23; *P* = 0.64; interaction: *F*_1.25,17.5_ = 0.11; *P* = 0.80) nor on the latency to pick up the reward from the food magazine on correct trials (log transformed; main effect: *F*_1,14_ = 1.30 *P* = 0.27; interaction: *F*_2,28_ = 1.79; *P* = 0.19). Finally, lesion did not affect the side bias (arcsine transformed; main effect: *F*_1,14_ = 0.14; *P* = 0.71; interaction: *F*_2,28_ = 2.44; *P* = 0.11; Table 2).

### Experiment 3: Effects of Selective 5-HT Depletion Within OFC and mPFC on Visual Discrimination Learning and Reversal

#### Neurochemical Results

**Figure 6:**
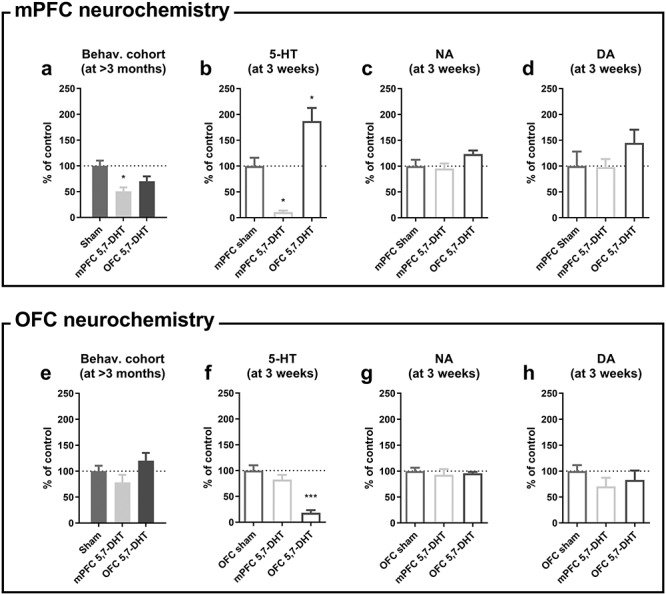
Serotonin (5-HT) levels recovered 3 months after 5,7-DHT infusions in the behavioral cohort (Expt. 3), but remained depleted 3 weeks after 5,7-DHT infusions in a control group (Expt. 4). 5-HT levels had partly and fully recovered in the behavioral cohorts approximately 3 months after mPFC (*a*) and OFC treatment (*e*), respectively. Three weeks after local 5,7-DHT infusions in the neurochemistry cohort, 5-HT levels were depleted by 90% in the mPFC (*b*) and 80% in the OFC (*f*), respectively. After OFC 5,7-DHT infusions, 5-HT levels in the mPFC were increased (*b*). No effect of 5,7-DHT treatment on either (*c*, *g*) noradrenaline or (*d*, *h*) dopamine in the mPFC or OFC in the neurochemistry cohort. ^*^P < 0.05 versus Sham; ^*^^*^^*^P < 0.001 versus Sham; one-tailed pairwise comparisons were used to detect 5-HT depletion in the target areas and two-tailed tests for all other comparisons.

In the behavioral cohort, killed >3 months after local 5,7-DHT infusions into either the mPFC or the OFC (see timeline in Fig. 1), 5-HT innervation of the prefrontal cortex had largely recovered, as indicated by no difference in 5-HT levels in the OFC group and a partial depletion in the mPFC group (see Fig. 6*a*,*e*). In contrast, in a control experiment, rats killed 3 weeks after 5,7-DHT infusions into the mPFC or OFC displayed significantly reduced 5-HT levels in the corresponding region (i.e., mPFC and OFC, respectively; Fig. 6*b*,*f*). Noradrenaline or dopamine levels were not affected (Fig. 6*c*,*d*,*g*,*h*), but note that there was an increase in mPFC 5-HT levels after OFC infusions of 5,7-DHT (Fig. 6*b*).

#### Behavioral Results

**Figure 7:**
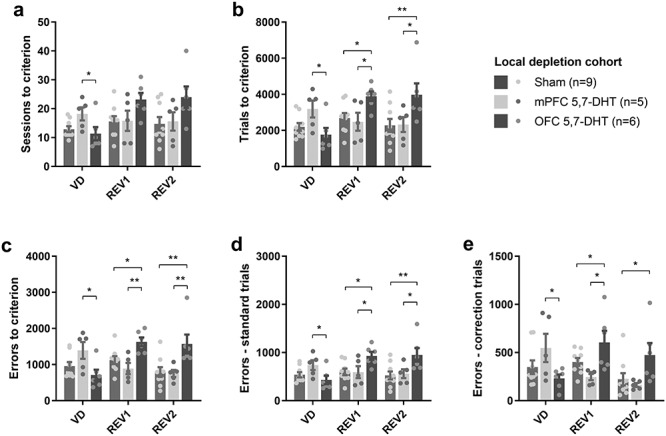
Effects of mPFC and OFC 5,7-DHT infusions on visual discrimination and reversal learning (Expt. 3). (*a*) 5-HT depletion of the mPFC impaired visual discrimination learning versus 5-HT depletion of the OFC, whereas the number of sessions needed to reach criterion in the reversal-learning stage was not significantly increased after OFC 5-HT depletion. (*b*) Trials committed before reaching criterion was increased during the visual discrimination stage after mPFC 5,7-DHT infusions (vs. OFC rats), whereas 5,7-DHT infusions into the OFC increased the trials to criterion during the reversal stages (vs. both sham controls and mPFC rats). Similar effects were noted on (*c*) total errors to criterion, (*d*) errors on standard trials, and (*e*) errors on correction trials. ^*^P < 0.05; ^*^^*^P < 0.01; Fisher’s LSD post hoc comparison after significant group × stage interaction and significant one-way ANOVA for the relevant stage.

When the mPFC and OFC were targeted by local infusions of 5,7-DHT, the effect on the number of sessions required to reach the 85% criterion depended both on the stage of testing and lesion group, as indicated by a stage × lesion interaction (square root transformed; *F*_4,34_ = 6.26; *P* = 0.001; Fig. 7*a*). This interaction was driven by different effects within the mPFC and OFC across the different stages: the lesion effect was significant during acquisition (one-way ANOVA: *F*_2,17_ = 3.68; *P* = 0.047) and failed to reach significance during reversal 1 (*F*_2,17_ = 2.90; *P* = 0.083) and reversal 2 (*F*_2,17_ = 2.72; *P* = 0.094). As can be seen in Figure 7*a*, the significant lesion effect during acquisition was mainly driven by an elevated number of sessions to criterion in the mPFC group. In pairwise comparisons, the mPFC rats needed more sessions to reach criterion during acquisition than did OFC rats (*P* = 0.017), whereas the numerical difference between mPFC rats and sham controls failed to reach significance (*P* = 0.058). There was similarly a stage × lesion interaction with regard to the number of trials required to reach criterion (square root transformed; *F*_4,34_ = 8.23; *P* < 0.001; Fig. 7*b*). In this case, the lesion effect was significant at each stage: acquisition (*F*_4,34_ = 4.16; *P* = 0.034), reversal 1 (*F*_4,34_ = 4.24, *P* = 0.032), and reversal 2 (*F*_4,34_ = 4.12; *P* = 0.035). Rats with mPFC depletions were significantly slower than rats with OFC depletions during acquisition (*P* = 0.011), but the numerical difference between mPFC rats and shams did not reach significance (*P* = 0.065). The OFC group was impaired versus both sham controls and the mPFC group during reversals 1 and 2 (Fig. 7*b*).

In agreement with the effects on session and trials, there was a significant stage × lesion interaction (square root transformed; *F*_4,34_ = 8.84; *P* < 0.001) on the total number of errors committed before the rats reached criterion (Fig. 7*c*). Breaking this effect down into the different stages, we found that one-way ANOVA were significant for each of the stages i.e., acquisition (*F*_2,17_ = 4.22; *P* = 0.033), reversal 1 (*F*_2,17_ = 7.45; *P* = 0.005) and reversal 2 (*F*_2,17_ = 6.69; *P* = 0.007). Specifically, rats with mPFC depletions committed more errors before reaching acquisition criterion than did rats with OFC depletions (*P* = 0.010) and there was a numerical but nonsignificant difference between the mPFC group and shams (*P* = 0.090). In contrast, OFC 5-HT depletion, but not mPFC 5-HT depletion, impaired reversal learning (*P* < 0.05; see Fig. 7*c*).

The same overall pattern of results was observed when the errors were analyzed separately on noncorrection trials and correction trials. On noncorrection trial errors, there was a significant stage × lesion interaction (square root transformed; *F*_4,34_ = 7.72; *P* < 0.001; Fig. 7*d*) and significant one-way ANOVA during acquisition (*F*_2,17_ = 3.67; *P* = 0.047), reversal 1 (*F*_2,17_ = 4.28; *P* = 0.031) and reversal 2 (*F*_2,17_ = 4.77; *P* = 0.023); on correction trials, there was similarly a significant stage × lesion interaction (log transformed; *F*_4,34_ = 7.44; *P* < 0.001; Fig. 7*e*) and significant one-way ANOVA for acquisition (*F*_2,17_ = 3.94; *P* = 0.039), reversal 1 (*F*_2,17_ = 3.76; *P* = 0.044) and reversal 2 (*F*_2,17_ = 4.30; *P* = 0.031).

Latencies to respond on correct and incorrect trials, as well as the latency to collect the reward from the food magazine after a correct response, were also analyzed, based on data from standard noncorrection trials only (Table 2). Correct latency was affected by lesion (*F*_2,17_ = 3.81; *P* = 0.043), but this effect did not vary across stages (lesion × stage interaction: *F*_4,34_ = 1.06; *P* = 0.39). Post hoc analysis revealed that mPFC 5,7-DHT treatment slowed down responses on correct trials (*P* = 0.013 vs. sham), whereas the OFC group was unaffected (*P* = 0.33 vs. sham). The latency to respond on incorrect trials was also affected by lesion (log transformed; *F*_2,17_ = 5.41; *P* = 0.005), without a significant lesion × stage interaction (*F*_4,34_ = 2.07; *P* = 0.11). Post hoc analysis showed that the mPFC group responded slower than both sham rats (*P* = 0.004) and rats from the OFC group (*P* = 0.048). Lesion group did not affect magazine latencies (*F*_2,17_ = 0.499; *P* = 0.62), and there was no lesion × stage interaction (*F*_4,34_ = 1.5; *P* = 0.22). There was also no main effect of lesion (*F*_2,17_ = 0.34; *P* = 0.15) or lesion × stage interaction (*F*_4,34_ = 0.045; *P* = 0.996) on side bias.

In summary, the experiment showed differential effects on visual discrimination and reversal learning after 5,7-DHT infusions in the OFC and mPFC. OFC infusions impaired performance during the reversal-learning stages but did not affect acquisition, whereas mPFC treatment increased response latencies regardless of stage and impaired acquisition compared to the OFC but not the sham control group, with no effect on reversal learning.

## Discussion

We attained selective 5-HT depletions globally and localized within the mPFC or OFC in rats through 5,7-DHT infusions combined with desipramine pretreatment to protect noradrenaline neurons. The 5-HT depletions differentially affected acquisition of a visual discrimination paradigm in touchscreen chambers and the subsequent reversal of the stimulus–reward contingencies. Specifically, global 5-HT depletion impaired both the acquisition and the reversal of the two-choice visual discrimination, whereas OFC depletion selectively impaired reversal learning without affecting initial discrimination performance. mPFC depletion, in contrast, slowed down responses to the visual stimuli overall and also impaired learning during initial acquisition compared with the OFC, but not the sham group, without affecting subsequent reversal. These results, together with previous evidence from OFC 5,7-DHT infusions in marmoset monkeys, confirm a role for orbitofrontal 5-HT in visual reversal learning across species and extend our knowledge of functional dissociations between subregions of the prefrontal cortex in cognitive functions. The findings also show that learning and reversal deficits following global 5-HT depletion can be related to loss of 5-HT in its prefronto-cortical terminal domains. Moreover, the behavioral disinhibition (faster response latencies) observed following global 5-HT loss can be dissociated from the specific effects of local 5-HT depletion on learning and reversal.

### Impaired Visual Discrimination and Reversal Learning after Global 5-HT Depletion

Although considerable cross-species evidence links 5-HT to reinforcement learning and cognitive flexibility, this is to our knowledge the first report of impaired touchscreen visual reversal learning in rodents after cortical 5-HT depletion. Acute tryptophan depletion in humans, which transiently lowers 5-HT levels throughout the brain (Young et al. 1999), can impair deterministic reversal ((Rogers et al. 1999), but see (Talbot et al. 2006)) and affect aspects of probabilistic reversal learning in humans (Murphy et al. 2002; Rogers et al. 2003). In rodents, global 5-HT depletion by i.c.v. 5,7-DHT infusions impairs probabilistic serial spatial reversal learning (Bari et al. 2010) and PCPA injections selectively impair the reversal-learning stage in bowl-digging tasks (Lapiz-Bluhm et al. 2009; Wallace et al. 2014). Conversely, reversal learning can be facilitated by augmenting 5-HT tone in experimental animals (Bari et al. 2010; Brigman et al. 2010; Barlow et al. 2015).

Nevertheless, the current data add to evidence supporting the view that globally reduced 5-HT affects general aspects of reinforcement learning rather than reversal learning, specifically. The deficits in learning after global 5-HT depletion were not limited to the reversal stage, as the rats were impaired already during the initial visual discrimination, and the number of errors committed on correction trials, which are the highest during the early reversal phase when the animals perseverate at the previously correct stimulus, were not significantly affected during any stage. This finding partially recapitulates work by Izquierdo and colleagues, who reported severe impairments in reward learning in a touchscreen task after putative 5-HT depletion by PCPA administration in rats (Izquierdo et al. 2012). Acute escitalopram treatment in healthy volunteers, which has been suggested to reduce cortical 5-HT levels by activating autoreceptors in the raphe nuclei (Nord et al. 2013), impaired initial discrimination learning but not the reversal stage in a probabilistic visual discrimination task (Skandali et al. 2018), whereas acute citalopram treatment impaired both the initial discrimination and the reversal phase in the same task in human volunteers (Chamberlain, Muller, et al. 2006b). Furthermore, homozygous carriers of the L′ form of the 5HTTLPR genetic variation of *SLC6A4* were more sensitive to negative feedback in a probabilistic visual discrimination and reversal task as measured by the global lose-shift rate, but did not differ from other genotypes with regard to selective aspects of reversal learning (den Ouden et al. 2013). In more recent work, Matias and colleagues used fiber photometry to measure the activity of 5-HT neurons in the dorsal raphe nucleus (DRN) of mice tested in a Pavlovian-conditioning task where stimulus-outcome contingencies were changed unexpectedly (Matias et al. 2017); increased activity of DRN 5-HT neurons was observed both after unexpected rewards (sugar delivery) and aversive outcomes (air puffs to the eye), as well as after neutral outcomes when a reward was expected (neither sugar nor air puff), again suggesting a more general role for 5-HT in flexible, associative learning.

### Selective Impairment of Reversal Learning after OFC 5,7-DHT

Localized 5-HT depletion of the OFC impaired reversal learning relative to sham animals and mPFC 5HT-depleted rats. In contrast, no difference was observed between the OFC group and sham rats during the initial visual discrimination phase. Such selective impairment in touchscreen visual reversal learning was previously observed in marmoset monkeys after 5,7-DHT infusions into the OFC (Clarke et al. 2004, 2007). Similarly, 5-HT markers including low 5-HT levels in the lateral and medial OFC were linked to inflexibility in a spatial reversal-learning task (Barlow et al. 2015), whereas the balance between OFC 5-HT and dopamine in the putamen was linked to reversal-learning aptitude in vervet monkeys (Groman et al. 2013). Taken together, this strongly suggests that a general role for orbitofronto-cortical 5-HT in cognitive flexibility is conserved across species. Our data also add to overwhelming evidence linking the OFC to visual reversal learning in humans (Rahman et al. 1999; Fellows and Farah 2003; Hornak et al. 2004), nonhuman primates ((Jones and Mishkin 1972; Dias et al. 1996; Groman et al. 2013); but see (Rudebeck et al. 2013; Chau et al. 2015)), rats (Chudasama and Robbins 2003; Hervig et al. 2020a) and mice (Graybeal et al. 2011; Brigman et al. 2013; Marquardt et al. 2017).

The effect of local 5,7-DHT depletion on reversal learning was limited to the OFC, as cognitive flexibility was intact in rats following mPFC 5-HT depletions. Touchscreen visual reversal learning is dependent on the integrity of the mPFC in rats and mice, as targeted lesions to the prelimbic cortex improved (Graybeal et al. 2011; McAllister et al. 2015), whereas selective lesions to the infralimbic cortex impaired later stages of the task (Chudasama and Robbins 2003). Early investigations also found that lesions including both infralimbic and prelimbic cortices impaired the late stage of visual reversal learning, but only when the stimuli were difficult to discriminate (Bussey et al. 1997). Pharmacological inactivation using baclofen/muscimol infusions into the infralimbic or prelimbic cortices more recently tended to improve serial visual reversal learning (Hervig et al. 2020a). Although caution must be applied when interpreting the data from our mPFC group due to low numbers, the data indicate that compromised mPFC 5-HT signaling through localized depletion does not affect reversal learning while significantly affecting some aspects of performance during initial learning.

### mPFC and Visual Discrimination Learning

5-HT depletion of the mPFC did not affect the reversal-learning stages, but the rats that had received mPFC infusions of 5,7-DHT performed worse during the initial acquisition than did the OFC rats. (There was a numerical, albeit nonsignificant, difference between mPFC-treated rats and sham controls during this stage.) A putative role for mPFC 5-HT in learning is supported by Masaki et al., who reported that 5-HT levels in the mPFC (as well as in the amygdala) of rats treated with parachloroamphetamine negatively correlated with discrimination learning (but also reversal performance) in a go/no-go task for rats (Masaki et al. 2006). In addition, Izquierdo and colleagues found that 5-HT content in the ventral prefrontal cortex (covering infralimbic and prelimbic cortex as well as the OFC) after PCPA treatment was inversely associated with the pretraining performance (reward learning) on a visual touchscreen task, i.e., low 5-HT levels were observed in slow learners (Izquierdo et al. 2012).

### Lack of Effect when 5,7-DHT Infusions were Made After the Initial Visual Discrimination

Global 5-HT depletion affected neither the retention nor the reversal of a visual discrimination that had been learned prior to the 5,7-DHT infusions. This result leads to two major conclusions. First, the effects of 5-HT depletion on visual discrimination acquisition were not caused by performance effects but more likely were on initial learning. Second, the lack of effect on reversal suggests that the effects of 5-HT depletion during initial learning impacts the reversal-learning stage when subjects are required to disengage from previously reinforced but now obsolete stimulus–reward contingencies. Consistent with this view, mice trained (drug-free) to criterion on a two-choice visual discrimination task in the touchscreen setting were not affected by the injection of PCPA prior to the first (and only) reversal of the stimulus–reward contingencies (Brigman et al. 2010). The effect of PFC 5-HT depletion in marmoset monkeys is only slightly different: although the 5,7-DHT infusions were made prior to the acquisition of the relevant stimulus–reward associations, 5-HT depletion did not affect the first reversal in a serial reversal task, but strongly impaired performance on the second and subsequent reversals (Clarke et al. 2004). Future work using reversible inactivation of 5-HT release in the OFC (e.g., using optogenetics) could clarify this issue.

### Neurochemistry: Recovery of 5-HT and Possible Compensatory Mechanisms

After local infusions of 5,7-DHT into the OFC and mPFC, the serotonergic innervations had undergone a full and partial recovery, respectively, in the behavioral cohorts, killed >3 months after surgery. Whereas we thus were unable to confirm that the animals had significant bilateral 5-HT depletion at each stage of the testing, brain samples collected from a separate cohort at a 3-week time point—corresponding to the beginning of the first reversal in the behavioral cohorts—showed profound and selective depletions of 5-HT in the target areas. These effects are in line with early evidence showing long-term re-growth of serotonergic fibers and recovery of function after 5,7-DHT infusions and similar treatments (Björklund et al. 1973; Wuttke et al. 1977; Frankfurt and Beaudet 1987). It therefore seems plausible that 5-HT fibers in the OFC and mPFC are damaged by the local 5,7-DHT infusions in the current experiments, but that the fibers regrow if allowed sufficient time. Recovery can be expected to be significantly slower, or simply not possible, after the i.c.v. infusion of 5,7-DHT, where the 5-HT cells themselves, and not just the axons, are affected by the toxin (Björklund et al. 1973; Wuttke et al. 1977; Wiklund et al. 1978). Due to the timeline of the recovery, and since the mPFC-treated rats from the behavioral cohort still presented with significant depletion in the mPFC, we conclude that 5-HT recovery was unlikely to have caused any behavioral effects (or lack of them) here.

The concept of re-growth of serotonergic fibers could also have contributed to the increased levels of 5-HT that we surprisingly observed in the mPFC after OFC 5,7-DHT infusions in the neurochemistry cohort. Individual 5-HT neurons can innervate multiple brain regions by wide-spread axonal arborization (Gagnon and Parent 2014); it is conceivable that the intact axon branches, for example those targeting neighboring areas such as the mPFC, develop a denser arbor structure in the process of re-growing fibers that have been lost in the OFC after 5,7-DHT infusions, leading to the observed increase in tissue content of mPFC 5-HT. It is also conceivable that the effect of elevated mPFC 5-HT following OFC 5-HT depletion is produced by altered activity of cortical projection neurons targeting the DRN, which could, in turn, increase the release of cortical 5-HT (in the mPFC, in this case). However, it must be noted that whereas pyramidal neurons in the prefrontal cortex have been shown to enhance the activity of 5-HT neurons in the DRN (Martin-Ruiz et al. 2001), such cortico-brainstem signaling was stimulated by cortical 5-HT_2A_ receptors (Martin-Ruiz et al. 2001; Puig et al. 2003), a finding which argues against a compensatory increase in mPFC 5-HT in the current experiment after OFC 5-HT depletion. Importantly, although we cannot rule out that the elevated mPFC 5-HT levels contributed to the behavioral impairments after OFC 5,7-DHT infusion, such a deleterious effect of prefrontal 5-HT seems unlikely, since reversal learning in marmoset monkeys is impaired after OFC 5-HT depletion that did not also lead to 5-HT up-regulation in the mPFC (Clarke et al. 2004, 2007).

Noradrenaline tissue content increased following i.c.v. 5,7-DHT administration, an effect which reached statistical significance in the OFC in the postacquisition cohort (Expt. 2). Monoaminergic brainstem nuclei including the DRN and the locus coeruleus innervate largely overlapping regions of the forebrain including the mPFC and OFC (Chandler et al. 2013), but are also anatomically interconnected (e.g., Pickel et al. 1977; Kim et al. 2004) and exert bidirectional control over one another (Pudovkina et al. 2002). Electrical stimulation of the DRN inhibits activity in the locus coeruleus, an effect which is abolished by 5,7-DHT pretreatment (Segal 1979), whereas lesions to the raphe nuclei as well as global 5-HT depletion increase the locus coeruleus levels of the noradrenaline-synthesizing enzyme tyrosine hydroxylase (McRae-Degueurce et al. 1982). It is plausible, therefore, that the loss of 5-HT innervation in the locus coeruleus after i.c.v. 5,7-DHT disinhibited noradrenaline synthesis and increased tissue content in efferent regions, notably the OFC, in the present experiments.

### Limitations

One limitation to bear in mind in particular when considering the effects of local 5-HT depletion on visual discrimination and reversal learning is that the final number of rats was quite low for the mPFC (*n* = 5) and the OFC (*n* = 6) groups. Whereas this was sufficient to detect the large impact of OFC depletion on reversal learning and effects on latencies of mPFC depletion, the effect on learning in the mPFC group was only statistically significant in comparison to the OFC lesioned rats; a future higher-powered experiment should further confirm the precise role of mPFC 5-HT in visual discrimination learning. Our methods also did not distinguish between subregions of the mPFC (e.g., prelimbic and infralimbic) or the OFC (medial vs. lateral) although such subregions could differentially contribute to reversal-learning performance according to some (Dalton et al. 2016; Hervig et al. 2020a) but not all investigations (Verharen et al. 2020). In addition, the current experiments did not address receptor specificity. Systemic 5-HT_2C_-receptor antagonism by SB-242084 impaired visual discrimination in mice (Phillips et al. 2018) as well as new learning (performance during the later phase) in a visual reversal-learning paradigm for rats (Alsiö et al. 2015). It is conceivable that loss of signaling at 5-HT_2C_ receptors contributes to the impairment observed here in visual discrimination after global 5-HT depletion. The impairment in reversal learning after OFC depletion is unlikely to be mediated by 5-HT_2C_ receptors, as antagonism of this receptor at the level of the OFC improves visual reversal learning (Alsiö et al. 2015), but it may implicate loss of activity at 5-HT_2A_ receptors, since reduced 5-HT_2A_ binding in the medial and lateral OFC has been linked to increased perseveration in a spatial reversal-learning task (Barlow et al. 2015) and since local infusions into the lateral OFC of a 5-HT_2A_ antagonist impaired serial visual reversal (Hervig et al. 2020b).

## Conclusion

Global 5-HT depletion by central infusions of 5,7-DHT impaired both the acquisition and subsequent reversal of a two-choice visual discrimination in rats, if the lesion was performed prior to the initial acquisition. In contrast, selective depletion of 5-HT from the OFC impaired reversal learning while leaving initial discrimination intact, whereas 5-HT depletion of the mPFC only marginally impaired reversal learning while slowing response latencies throughout. These sub-region specific effects add to our understanding of the role of 5-HT in reinforcement learning and cognitive flexibility. Future work focusing on the precise 5-HT receptors involved could lead to the development of better pharmacotherapies for disorders such as obsessive–compulsive disorder.

## Funding

Wellcome Trust Senior Investigator (award 104631/Z/14/Z to T.W.R.). All experiments were conducted at the University of Cambridge Behavioral and Clinical Neuroscience Institute, which was jointly funded by the Medical Research Council and the Wellcome Trust.

## Notes

We are grateful to Dr Simon R.O. Nilsson for comments on an earlier version of the manuscript. Part of the experiments were carried out under a Home Office Project Licence held by Dr A.L. Milton.

## Conflict of interest

T.W.R. is a consultant for, and receives royalties from, Cambridge Cognition; is a consultant for Unilever and Greenfield Bioventures, had recent research grants with Shionogi and Small Pharma and GlaxoSmithKline and receives editorial honoraria from Springer Nature and Elsevier. J.W.D. had recent grants from GlaxoSmithKline and Boehringer Ingelheim and receives editorial honoraria from the British Neuroscience Association. The rest of the authors declare no conflict of interest.
